# *Cryptosporidium parvum* competes with the intestinal epithelial cells for glucose and impairs systemic glucose supply in neonatal calves

**DOI:** 10.1186/s13567-023-01172-y

**Published:** 2023-05-03

**Authors:** Franziska Dengler, Harald M. Hammon, Wendy Liermann, Solvig Görs, Lisa Bachmann, Christiane Helm, Reiner Ulrich, Cora Delling

**Affiliations:** 1 Institute of Physiology, Pathophysiology and Biophysics, University of Veterinary Medicine Vienna, Veterinärplatz 1, Vienna, Austria; 2 Institute of Veterinary Physiology, Leipzig University, An den Tierkliniken 7, Leipzig, Germany; 3 Research Institute for Farm Animal Biology (FBN), Institute of Nutritional Physiology, Wilhelm-Stahl-Allee 2, Dummerstorf, Germany; 4 Faculty of Agriculture and Food Science, University of Applied Science Neubrandenburg, Brodaer Strasse 2, Neubrandenburg, Germany; 5 Institute of Veterinary Pathology, Leipzig University, An den Tierkliniken 33-37, Leipzig, Germany; 6 Institute of Parasitology, Leipzig University, An den Tierkliniken 35, Leipzig, Germany

**Keywords:** Cryptosporidiosis, GLUT2, in vivo, metabolism, SGLT1, Ussing chamber

## Abstract

Cryptosporidiosis is one of the main causes of diarrhea in children and young livestock. The interaction of the parasite with the intestinal host cells has not been characterized thoroughly yet but may be affected by the nutritional demand of the parasite. Hence, we aimed to investigate the impact of *C. parvum* infection on glucose metabolism in neonatal calves. Therefore, *N* = 5 neonatal calves were infected with *C. parvum* on the first day of life, whereas a control group was not (*N* = 5). The calves were monitored clinically for one week, and glucose absorption, turnover and oxidation were assessed using stable isotope labelled glucose. The transepithelial transport of glucose was measured using the Ussing chamber technique. Glucose transporters were quantified on gene and protein expression level using RT-qPCR and Western blot in the jejunum epithelium and brush border membrane preparations. Plasma glucose concentration and oral glucose absorption were decreased despite an increased electrogenic phlorizin sensitive transepithelial transport of glucose in infected calves. No difference in the gene or protein abundance of glucose transporters, but an enrichment of glucose transporter 2 in the brush border was observed in the infected calves. Furthermore, the mRNA for enzymes of the glycolysis pathway was increased indicating enhanced glucose oxidation in the infected gut. In summary, *C. parvum* infection modulates intestinal epithelial glucose absorption and metabolism. We assume that the metabolic competition of the parasite for glucose causes the host cells to upregulate their uptake mechanisms and metabolic machinery to compensate for the energy losses.

**Supplementary Information:**

The online version contains supplementary material available at 10.1186/s13567-023-01172-y.

## Introduction

The protozoan parasite *Cryptosporidium parvum* has been identified as one of the top four pathogens causing diarrhea in young children in Asia and Africa [1, 2]. Apart from human infection, *C. parvum* is a leading cause of diarrhea in calves and thus impairs animal welfare and infers immense economic losses in calf rearing worldwide [3–5]. It infects the epithelial cells of the gastrointestinal tract, especially the distal parts of the small intestine, and reproduces in the enterocytes [6]. After infection, *C. parvum* completes its whole life cycle, consisting of an asexual and a sexual part, in the same host [7]. The newly shed oocysts, which have a high tenacity in the environment, can infect the next suitable host directly, leading to clinical signs of varying severity, particularly diarrhea. In immunocompetent hosts, this acute gastroenteritis is self-limiting but children under two years of age or immunocompromised patients such as HIV patients are more often affected severely and the disease may be chronic or even fatal [8]. So far, there is no fully effective therapy available, which is at least partly due to lacking understanding of the pathophysiology [9, 10].

There is accumulating evidence that *C. parvum* relies heavily on nutrients supplied by the host cell. Being encapsulated in a parasitophorous vacuole, the parasite is localized intracellularly but extracytoplasmatic and thus escapes cellular defense mechanisms [11]. The host-parasite interface forms a so called “feeder organelle”, an electron dense membrane with a highly increased surface area that is postulated to mediate the uptake of nutrients from the host cell’s cytoplasm into the parasitophorous vacuole [12]. In the course of parasitic evolution, the parasite has lost most of its machinery for efficient (i.e., oxidative) energy production with glycolysis as the only remaining pathway [13]. An increased rate of glycolysis has been observed in in vitro models of *C. parvum* infection [14, 15]. It is thus speculated, that specific targeting of the parasitic glycolysis might be a promising therapeutic approach [16–18]. However, the premise for a specific intervention is a thorough understanding of the host cells’ metabolic adaptation. Yet, most studies on the pathophysiology of cryptosporidiosis so far have been conducted in vitro using a cell culture model, where the *C. parvum* reproductive cycle cannot be completed, or in rodent models, which might not show the same pathophysiological reaction as the natural hosts [9]. It is therefore not clear, whether the in vitro findings can be transferred to the in vivo situation of calves and how the intact intestinal epithelium reacts to infection and nutritional competition.

Physiologically, the enterocytes of the small intestine efficiently take up glucose from the gut lumen. This is mediated by a secondary active transport mechanism, a Na^+^/glucose-symporter (SGLT1), and is therefore independent of a concentration gradient for glucose [19]. Although glucose is one of the main fuels for the enterocytes themselves, most of the glucose taken up from the gut lumen is further transported into the splanchnic circulation. This export is mediated by glucose transporter (GLUT) 2, which mediates the facilitated diffusion of glucose [20]. This strict separation between apical and basolateral transport mechanisms has been demonstrated to be corrupted under various conditions including infectious diseases, stress or high luminal glucose concentrations [21–23]. It was postulated that GLUT2 is recruited to the apical membrane to enhance cellular glucose supply under these conditions, but this concept is controversial [20, 24]. In IPEC-J2 cells infected with *C. parvum*, we found a displacement of GLUT2 from the cell membrane into the cytoplasm [14], indicating that the parasite interferes with epithelial glucose transport actively or passively. With the parasite relying on cellular glucose supplies, it might have developed strategies to prevent the export of glucose from the enterocytes into the circulation just as it has been demonstrated to regulate cellular apoptosis [25]. However, a reorganization of the transepithelial glucose transport machinery could also reflect the host’s attempt to rescue the cellular and systemic energy supply given the nutritional competition of the parasite. Additionally, intestinal epithelial glucose transport was postulated to have a role in host immune defense and inflammatory diseases [22]. To date, this is speculative, as the metabolism of glucose during *C. parvum* infection has not been investigated in vivo yet. Therefore, we aimed to investigate the effects of infection with *C. parvum* in neonatal calves on the transepithelial transport and systemic glucose metabolism. We hypothesized that *C. parvum* infection results in impaired transepithelial glucose uptake by influencing the cellular transport machinery directly or indirectly in favor of high glucose levels within the enterocytes, i.e., leading to an increased uptake via SGLT1 and a reduced export via GLUT2.

## Materials and methods

### Animals

Ten male neonatal calves (*bos taurus*, Holstein-Friesian) were used. After birth, the calves were fed 3 L of colostrum on the farm. The colostrum was pooled before the beginning of the experiment and stored frozen in portions, so that all animals received the same colostrum. Only calves that consumed the whole 3 L and were clinically healthy were included in the study. Then, the calves were transported to the University of Leipzig within their first 24 h of life. There, they were stalled in one of two separate barns (control and infection) on wood shavings with only one calf concurrently. The calves were fed 3 × 2 L milk replacer (Union A50 S, Arla Foods, Viby J, Denmark) daily and had free access to water in a bucket. Additionally, they received 2 L electrolytes (Ursolyt G oral, Serumwerk Bernburg AG, Bernburg, Germany) daily from day 4 post-infection (pi). The intake of the oral rehydration solution was documented.

Upon arrival at the University (= day 0) the animals were examined clinically, and a blood sample was collected. Afterwards, one group of calves was infected by oral application of 2 × 10^7^* C. parvum* oocysts whereas the control group received pure water. In the following days, the calves were monitored clinically, fecal shedding of *C. parvum* oocysts was quantified and fecal consistency was assessed using a score from 0 to 3 (0 = normal, 1 = mushy, 2 = liquid, 3 = liquid with blood addition). For quantification, the commercial immunofluorescence assay kit MERIFLUOR^®^ Cryptosporidium/Giardia (Meridian Bioscience, Inc., Cincinnati, USA) was used according to the manufacturer’s protocol with a standardized amount of feces sample as part of a standard operation procedure of the Institute for Parasitology. Additionally, fecal samples were tested for *E. coli* K99, Rota- and Coronavirus on day 0 and 7 using a snap test (BoDia, Fassisi, Göttingen, Germany).

Furthermore, EDTA and Li-Heparin blood samples were taken on days 0, 4, 6 and 7 pi. On day 6 pi, the glucose metabolism was assessed using a tracer technique (see below) and on day 7 the animals were anaesthetized with thiopental (Thiopental Inresa 0.5 g, 30–40 mg kg^− 1^ i.v. by effect, Inresa Arzneimittel GmbH, Freiburg, Germany) and killed by exsanguination through the opening of both *Vv. jugulares*. Then, the abdominal cavity was opened and samples for subsequent analyses were taken as described below. The downstream experiments focused on the distal jejunum epithelium, as this is both a predominant site of infection and highly active in glucose absorption physiologically.

The experiments were conducted in accordance with the German legislation on the protection of animals and were licensed by the Landesdirektion Leipzig as TVV 19/20.

### *C. parvum*

The in-house strain of *C. parvum* (LE-01-Cp-15) was used in all experiments. It was thoroughly characterized previously [26, 27]. The strain was passaged regularly in neonatal calves, and freshly recovered oocysts were isolated and stored as described earlier [28, 29]. The same batch was used for all experiments. Before use, oocysts were washed with phosphate buffered saline (PBS) once and centrifuged at 6000 ×*g* for 8 min. Then the oocysts were resuspended in PBS and counted in a Neubauer chamber.

### Autopsy and histopathology

After euthanasia on day 7 pi, an autopsy was performed, including standardized sampling of the gastrointestinal tract for histopathology, immunohistochemistry and further examinations. Macroscopically, the grade of catarrhal enteritis was scored based on the degree of liquefaction of the ingesta in the intestinal tract as follows: 0 = no enteritis, 1 = mild enteritis, 2 = moderate enteritis, 3 = severe enteritis. Specimen for histopathology were fixed in 4% neutral-buffered formaldehyde, processed, embedded in paraffin and 3 μm thin slices were stained with hematoxylin and eosin. Light microscopic examination was performed using an Olympus BX53 light microscope (Olympus, Shinjuku, Japan). An Olympus DP26 color digital camera and cellSens imaging software (Olympus, Shinjuku, Japan) were used for digital photography and measurements of the length of crypts and villi.

### Blood chemistry

Plasma concentrations of glucose and urea were determined using an automatic spectrophotometer (ABX Pentra 400; Horiba ABX SAS, Montpellier, France) with kits from MTI Diagnostics (Idstein, Germany; glucose: 5530230) and Labor + Technik E. Lehmann (Berlin, Germany; urea: LT UR0010). Intra-assay coefficients of variation were below 0.1% for all parameters.

### Tracer study

On day 6 pi, the appearance rate of orally and systemically administered glucose was monitored by application of [^13^C_6_]-labelled glucose orally (10 mg kg^− 1^, 99.10 atom% ^13^C, Cambridge Isotope Laboratories, Inc., Andover, MA, USA) with the morning feed and [6,6-^2^H_2_]-labelled glucose dissolved in 10 mL 0.9% NaCl i.v. (5 mg kg^− 1^, 99.36 atom% ^2^H, Campro Scientific GmbH, Berlin, Germany) simultaneously. Then, blood samples were collected from the *V. jugularis* over 8 h (at −10, −5, 0, 5, 10, 15, 20, 30, 45, 60, 90, 120, 150, 180, 210, 240, 270, 300, 330, 360, 420, 480 min from the application of tracers) and stored at −20 °C to measure ^13^C and ^2^H enrichment in plasma glucose using mass spectrometry as described previously [30–32]. Enrichment data were used to calculate the rate of appearance (Ra) of glucose in plasma with the following calculation:

$${\text{R}}{{\text{a}}_{{\text{i.v.}}}}\left[ {{\text{mmol }} \times {\text{k}}{{\text{g}}^{ - {\text{1}}}} \times {{\text{h}}^{ - {\text{1}}}}} \right]{{ = }}{{\text{D}}_{{\text{i.v.tracer}}}} \times {\text{ AUC}}\left( {\left[ {{}^{\text{2}}{{\text{H}}_{\text{2}}}} \right]{\text{-Glc}}} \right){\text{ and}}$$

$${\text{R}}{{\text{a}}_{{\text{oral}}}}\left[ {{\text{mmol }} \times {\text{ k}}{{\text{g}}^{ - {\text{1}}}} \times {{\text{h}}^{ - {\text{1}}}}} \right]{ = }{{\text{D}}_{{\text{oral tracer}}}} \times {\text{ AUC}}\left( {\left[ {{\text{13}}{{\text{C}}_{\text{6}}}} \right]{\text{-Glc}}} \right)$$

where D_i.v.tracer_ and D_oral tracer_ are the doses of labelled glucose (mmol kg^− 1^ BW) injected i.v. or administered orally, respectively, and AUC is the area under the glucose (Glc) enrichment–time curves (mole percent excess x min).

^13^CO_2_ enrichment in blood as a reliable indicator for glucose oxidation was analyzed as described by Gruse et al. [30].

### Ussing chamber analysis

The distal jejunum was excised and washed in 4 °C oxygenated buffer solution (see below). Then, the epithelium was manually stripped off the underlying muscle and the isolated epithelium was mounted in Ussing chambers as described previously [33]. The area exposed accounted for 1.1 cm^2^. Before the beginning of the experiment, the epithelia were allowed to equilibrate in the system for at least 30 min.

The buffer solution consisted of 120 mmol L^− 1^ NaCl, 5.5 mmol L^− 1^ KCl, 1.25 mmol L^− 1^ CaCl_2_, 1.25 mmol L^− 1^ MgCl, 0.6 mmol L^− 1^ NaH_2_PO_4_, 2.4 mmol L^− 1^ Na_2_HPO_4_, 10 mmol L^− 1^ glucose, 5 mmol L^− 1^ L-glutamine, and 10 mmol L^− 1^ HEPES and was used for rinsing, preparation, transport and incubation of the epithelia. For the glucose free buffer solution, glucose was replaced with mannitol, which was also used to adjust osmolarity to 280 mosmol L^− 1^. The pH was adjusted to 7.4 using HCl or NaOH. All buffer solutions were gassed with 100% oxygen (Linde Gas, Germany). All chemicals were obtained from Merck (Germany), Carl Roth (Germany) or VWR (Germany).

Electrical measurements were taken continuously with the aid of a computer-controlled voltage clamp device (Ingenieurbüro für Mess- und Datentechnik, Dipl.-Ing. K. Mußler, Aachen, Germany). All experiments were conducted under short-circuit conditions. The short-circuit current (I_sc_) and transepithelial tissue conductance (G_t_) were calculated computationally as described [34]. The different treatments in each experiment were assigned to the individual epithelia within one animal according to their G_t_ so that at the end of an experimental series, the mean value of G_t_ was similar in all treatment groups.

The electrogenic transport of glucose by SGLT1 was measured as the phlorizin sensitive increase in I_sc_ (ΔI_sc_) after a mucosal addition of glucose to the buffer solution repeatedly. Therefore, we incubated the epithelia in a glucose-free buffer solution on the mucosal side and measured ΔI_sc_ after the mucosal addition of 2 mmol L^− 1^ glucose in the following 10 min. Then, the buffer solution was replaced by a glucose-free buffer solution again and the measurement of ΔI_sc_ was repeated every 30 min four times. One group of epithelia (*n* = 3 technical replicates) was incubated with 0.2 mmol L^− 1^ phlorizin 20 min before the first addition of glucose, while others (*n* = 3) only received the solvent ethanol. The mean ΔI_sc_ of all technical replicates was calculated for each time point and we calculated the phlorizin sensitive I_sc_, i.e., the activity of SGLT1from the difference in glucose-induced ΔI_sc_ in the groups incubated with and without phlorizin.

### Two step RT-qPCR

Total RNA was isolated from 20 mg of the tissue which was homogenized in lysis buffer from the ReliaPrep™ RNA Miniprep System (Promega GmbH, Mannheim, Germany) using a FastPrep 24-5G (MP Biomedicals, Eschwege, Germany). The samples were further processed according to the manufacturer’s protocol including treatment with DNase. The RNA concentration and quality were determined using a spectrophotometer (DeNovix DS-11, Wilmington, USA). 1 µg of high-quality RNA was used for cDNA synthesis using the GoScript™ Reverse Transcriptase Kit (Promega, Mannheim, Germany) according to the manufacturer’s instructions using a GeneAmp 9700 PCR System (Applied Biosystems, Thermo Fisher Scientific, Vienna, Austria).

For qPCR, the resulting cDNA was diluted 1:10 and 1 µL was used in a 20 µL reaction volume containing a ready-to-use premix of SYBR Green I dye, dNTPs, stabilizers, and enhancers (GoTaq^®^, Promega GmbH, Mannheim, Germany), 112 nmol L^− 1^ primer mix and DNase-free water. These mixtures were pipetted in strip tubes (LabConsulting.at, Vösendorf, Austria) and processed in a Corbett Rotor-Gene 6000 (Qiagen, Hilden, Germany) at individually optimal protocols (Table 1). A no-template control (NTC) with DNase-free water instead of cDNA was applied for each run. qPCR reactions for each sample and gene were run in duplicate to minimize dispensation artefacts. The PCR cycles were run using automatic fluorescence emission following each PCR cycle, and the amplification specificity was checked after each run by melting curve analysis. The primer sequences for qPCR are shown in Table 1; the denaturation temperature was always 95 °C and extension and annealing were performed at 60 °C. The primers were designed with the Primer BLAST tool from the National Center for Biotechnology Information (NCBI, Bethesda, MD, USA) according to known sequences from the Basic Local Alignment Search Tool (BLAST) in the gene bank database of the NCBI and synthesized by Eurofins MWG (Ebersberg, Germany). The quantification cycle and amplification efficiency of each amplification curve were determined using the rotor gene 6000 Series Software 1.7 (Corbett/Qiagen, Hilden, Germany). The C_t_ values set by the software were applied after checking them optically. For analysis of the data, the ΔΔC_t_ method was used to compare the mRNA expression. Normalization of the samples was achieved using the same amounts of tissue and RNA for processing and by normalizing the data for the target genes with the aid of the reference genes β-actin (*bACTIN*), ribosomal protein L4 (*RPL4*), *RPL32*, peptidylprolyl-isomerase A (*PPIA*) and tyrosine 3-monooxygenase/tryptophan 5-monooxygenase activation protein zeta (*YWAHZ*). Therefore, the geometric mean of all reference genes’ C_t_ values was calculated for each sample and used for normalization. The reference genes have been tested for their stability under the experimental conditions applied in our study using the program RefFinder [35].

**Table 1:** **Primers used for RT-qPCR**

Gene name	Gene bank accession no.	Primer sequence (5′ – 3′)	Amplicon length (bp)
*bACTIN*	NM_173979.3	F: AGCCTTCCTTCCTGGGCAT	93
		R: TAGAGGTCCTTGCGGATGTC	
*GLUT1*	NM_174602.2	F: CGGCATCAACGCTGTTTTCT	105
		R: GAAGGCTGTGTTGACGATGC	
*GLUT2*	XM_005201668.4	F: GGTTCATGGTGGCTGAGTTT	161
		R: AGACCACACCAGCAAAAAGG	
*HK1*	NM_001012668.2	F: AGCTTCATCCGCACTTCTCC	96
		R: CCGCTGCCGTCTTCAGATAA	
*HK2*	XM_015473383.2	F: ATGGGCATGAAGGGTGTGTC	89
		R: CTTCAGGAGGATGCTCTCGTC	
*PFKl*	NM_001080244.2	F: ATCGTCATGTGCGTCATCCC	110
		R: GATGCGGTCACAACTCTCCAT	
*PPIA*	NM_178320.2	F: GGTCCTGGCATCTTGTCCAT	96
		R: GCTTGCCATCCAACCACTCA	
*RPL4*	NM_001014894.1	F: GCTCCCATTCGACCCGATATT	104
		R: AGCACTGGTTTGATGACCTGC	
*RPL32*	NM_001034783.2	F: AGACCCCTCGTGAAGCCTAA	95
		R: CCGCCAGTTCCGCTTGATTT	
*SGLT1*	NM_174606.2	F: TACATTAAGGCGGGGGTGGT	91
		R: AGGGACAGGACAGACAGGTA	
*YWAHZ*	NM_174814.2	F: ACCTACTCCGGACACAGAACA	89
		R: TCATCATATCGCTCAGCCTGC	

### Western blot

Total protein extracts and brush border membranes (BBM) were prepared from isolated jejunum mucosa samples that were snap frozen directly after sampling. Frozen tissue samples were minced and then thawed and homogenized in ice-cold buffer solution (2 mmol L^− 1^ Tris base, 50 mmol L^− 1^ mannitol, 0.1 mmol L^− 1^ PMSF, 5 mmol L^− 1^ EGTA; pH 7.1). From this homogenate, a first sample (P1) was collected and protease and phosphatase inhibitor was added (Halt™ Protease Inhibitor Cocktail, Thermo Fisher Scientific, Karlsruhe, Germany) to use it for analysis of total protein abundance. BBM were isolated by CaCl_2_ precipitation. After addition of CaCl_2_ to a final concentration of 10 mmol L^− 1^ and 20 min incubation while stirring at 4 °C, the suspension was centrifuged (2000*g*, 20 min, 4 °C). Then, the supernatant was centrifuged (25 000 *g*, 30 min, 4 °C) and the pellet was resuspended again in homogenization buffer and the previous steps were repeated once again. Subsequently, the final pellet was resolved in buffer (10 mmol L^− 1^ Tris base, 150 mmol L^− 1^ NaCl; pH 7.4) with a protease and phosphatase inhibitor (Halt™ Protease Inhibitor Cocktail) and stored at −20 °C (P2). The protein concentration was measured with the EnSpire Multimode Plate Reader (PerkinElmer LAS -GmbH, Rodgau, Germany) using the bicinchoninic acid method and bovine serum albumin as standard.

For Western blot analysis, the protein samples were separated by sodium dodecyl sulphate-polyacrylamide gel electrophoresis (SDS-PAGE) using 20 µg protein/well. Subsequently, the samples were transferred onto a nitrocellulose membrane (Nitropure 0.45 μm, Osmonics, Westborough, USA) using the EasyPhor Semi Dry Blotter (Biozym, Vienna, Austria). The membrane was preincubated in EveryBlot Blocking Buffer (Bio-Rad, Feldkirchen, Germany) for one hour. Then it was incubated with the primary antibodies (see Table 2) at 4 °C overnight with gentle agitation. After washing with TBST, the membranes were incubated with an HRP-coupled secondary antibody (see Table 2) at room temperature with gentle agitation for one hour. Subsequently, the membranes were rinsed again with TBST, then the signal was detected by enhanced chemiluminescence using a ChemiDoc IT 600 Imaging System (UVP/Analytik Jena GmbH, Jena, Germany) and analyzed using ImageJ (Rasband, W.S., ImageJ, U. S. National Institutes of Health, Bethesda, Maryland, USA). The blots were normalized to total protein content measured with the Pierce™ Reversible Protein Stain Kit for Nitrocellulose Membranes (Thermo Fisher Scientific, Karlsruhe, Germany). The enrichment of SGLT1, GLUT2 and VILLIN in the BBM preparations was calculated as the ratio of P2/P1, i.e., the protein abundance in BBM relative to the total protein content of the tissues.

**Table 2:** **Antibodies used for Western blot**

Primary antibody	Dilution	Distributor/catalogue no.	Secondary antibody	Dilution	Distributor/catalogue no.
Rabbit-anti-GLUT2	1:1000	Aviva Systems biology/#ARP41706_P050	Donkey-anti-rabbit HRP	1:10 000	Cellsignalling/#7074
Rabbit-anti-SGLT1	1:1000	Bioss Antibodies/#bs-1128R-TR			
Rabbit-anti-VILLIN	1:1000	Thermo Fisher Scientific/#PA5-78222			

### Immunofluorescent staining

For immunofluorescent staining of the glucose transporters, the tissues were fixed in 4% neutral-buffered formaldehyde, embedded in paraffin, and cut in 6 μm thin slices. After deparaffinization, antigen demasking with proteinase K and blocking, the slides were stained with primary antibody (see Table 3) overnight, rinsed with PBS and incubated with secondary antibody for one hour. The cell nuclei were counterstained with 4′,6-diamidino-2-phenylindole (DAPI, Merck, Darmstadt, Germany) and the parasites were stained using Sporo-Glo™ (A600Cy3R-1X, Waterborne, Inc., New Orleans, USA). The staining was documented using laser scanning confocal microscopy (LSM 880 and ZEN software, Carl Zeiss AG, Oberkochen, Germany).

**Table 3:** **Antibodies used for IF staining**

Primary antibody	Dilution	Distributor/catalogue no.	Secondary antibody	Dilution	Distributor/catalogue no.
Rabbit-anti-GLUT2	1:100	Abcam/#ab54460	Goat-anti-rabbit ALEXA 488	1:500	Thermo Fisher Scientific/#A-11,034
Rabbit-anti-SGLT1	1:100	Antibodies online/ABIN364451			

### Statistics

The results are shown and described as median, 10th, 25th, 75th and 90th percentiles with error bars or arithmetic means ± standard deviation (SD). The significance is expressed as the probability of error (p). Technical replicates were pooled for each animal (*N*) for statistical analysis. Statistical analysis was performed with SigmaPlot 14.5 (Systat Software, Erkrath, Germany). The data were checked for normality and equal variance using a Shapiro-Wilk and Brown-Forsythe test, respectively. Statistical differences were analyzed using a Student’s *t*-test or one- or two-Way Repeated Measurements (RM) ANOVA with post hoc Holm-Sidak test, Mann-Whitney Rank Sum Test or an ANOVA on ranks with post hoc Tukey test as appropriate. The differences were assumed to be statistically significant if *p* < 0.05.

## Results

### Feces score, oocyst shedding, body weight and internal body temperature

**Figure 1:**
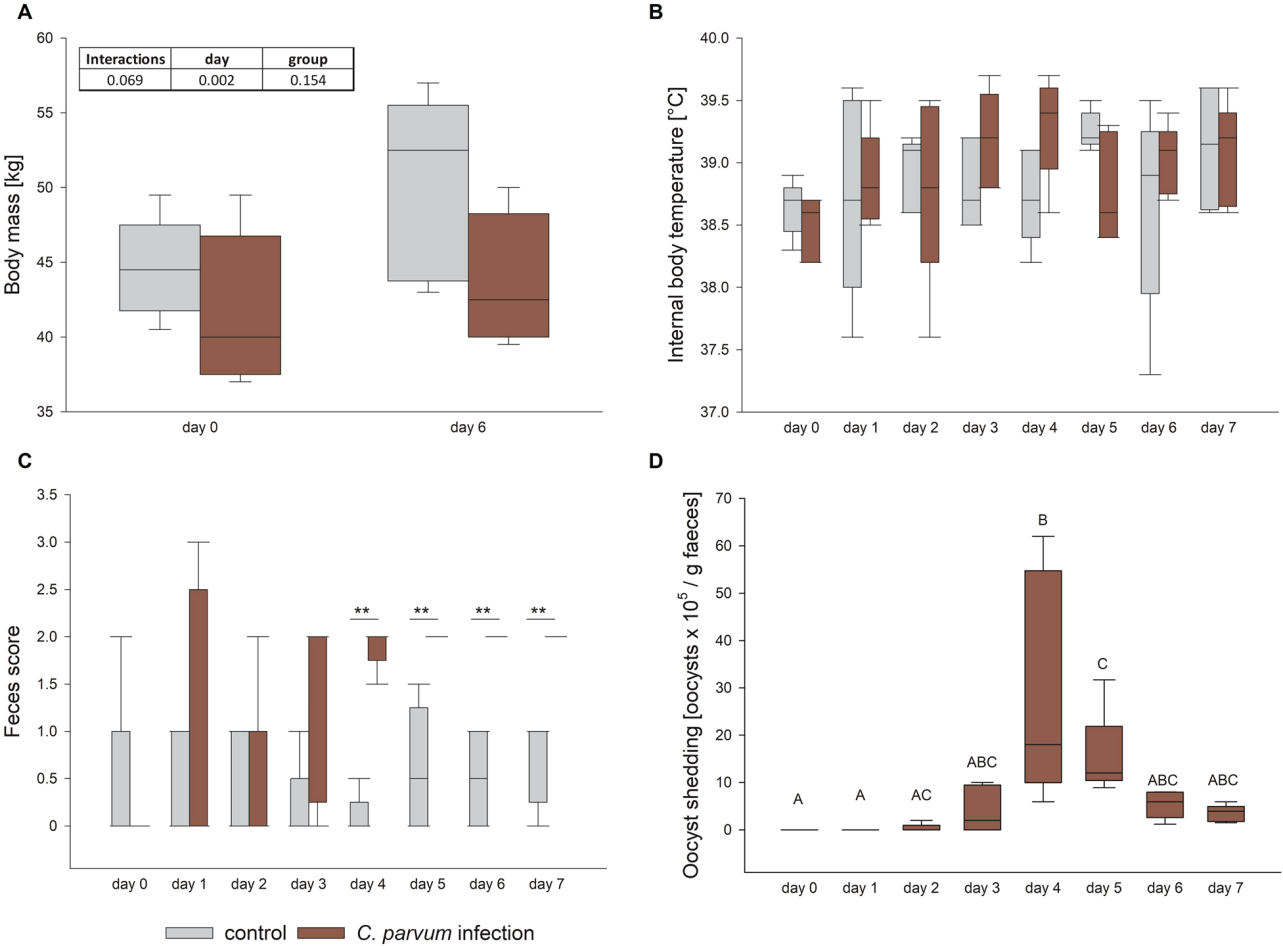
**Body weight (A), internal body temperature (B), fecal consistency (C) and oocyst shedding (D) in control (gray boxes) and infected calves (red boxes).** **A** The body weight was not different between the groups on day 0 and 6 pi but increased significantly over time. **B** The internal body temperature was not different between the groups or time points. **C** Fecal samples were scored from 0 to 3 (0 = normal, 1 = mushy, 2 = liquid, 3 = liquid with blood addition) and compared between the groups on each day, resulting in a significantly higher score in the infected calves from day 4 pi. **D** Oocyst shedding culminated on day 4 pi. Boxes show median and percentiles plus error bars; *N* = 5, different letters (A, B, C) indicate significant differences between different time points within the same group, two-way RM ANOVA with post-hoc Holm-Sidak-test (A, B) and RM ANOVA on ranks with post-hoc Tukey test (D), respectively, *p* < 0.05. Asterisks indicate significant differences between the groups, Mann-Whitney Rank Sum Test, **p < 0.01 (C).

The calves had a starting body weight of 44.6 ± 3.3 kg in the control and 41.7 ± 5.1 kg in the infection group, respectively. On day 6 pi, they had reached 50.2 ± 6.1 kg and 43.8 ± 4.4 kg, respectively. The interaction between the factors time and treatment group did not reach statistical significance (*p* = 0.069), but there was a significant increase from day 0 to 6 pi overall (Figure 1A; Additional file 1; *p* < 0.05, two-way RM ANOVA). There was no interaction and no difference regarding time point and treatment group for internal body temperature (Figure 1B; Additional file 1). The infected calves had diarrhea compared to mostly normal feces in the control group. This became obvious in significantly higher feces scores in the infected calves compared to the control group from day 4 pi (Figure 1C; Additional file 1; *p* < 0.01, Mann-Whitney Rank Sum Test). Similarly, the fecal shedding of oocysts by the infected calves increased from day 0 to day 4 pi, where it peaked and then declined again (Figure 1D; Additional file 1; *p* < 0.05, RM ANOVA on ranks with post-hoc Tukey test). The control animals did not shed *C. parvum* oocysts and all calves were free of *E. coli* K99, Rota- and Coronavirus.

### Macroscopical and histopathological examinations

**Figure 2:**
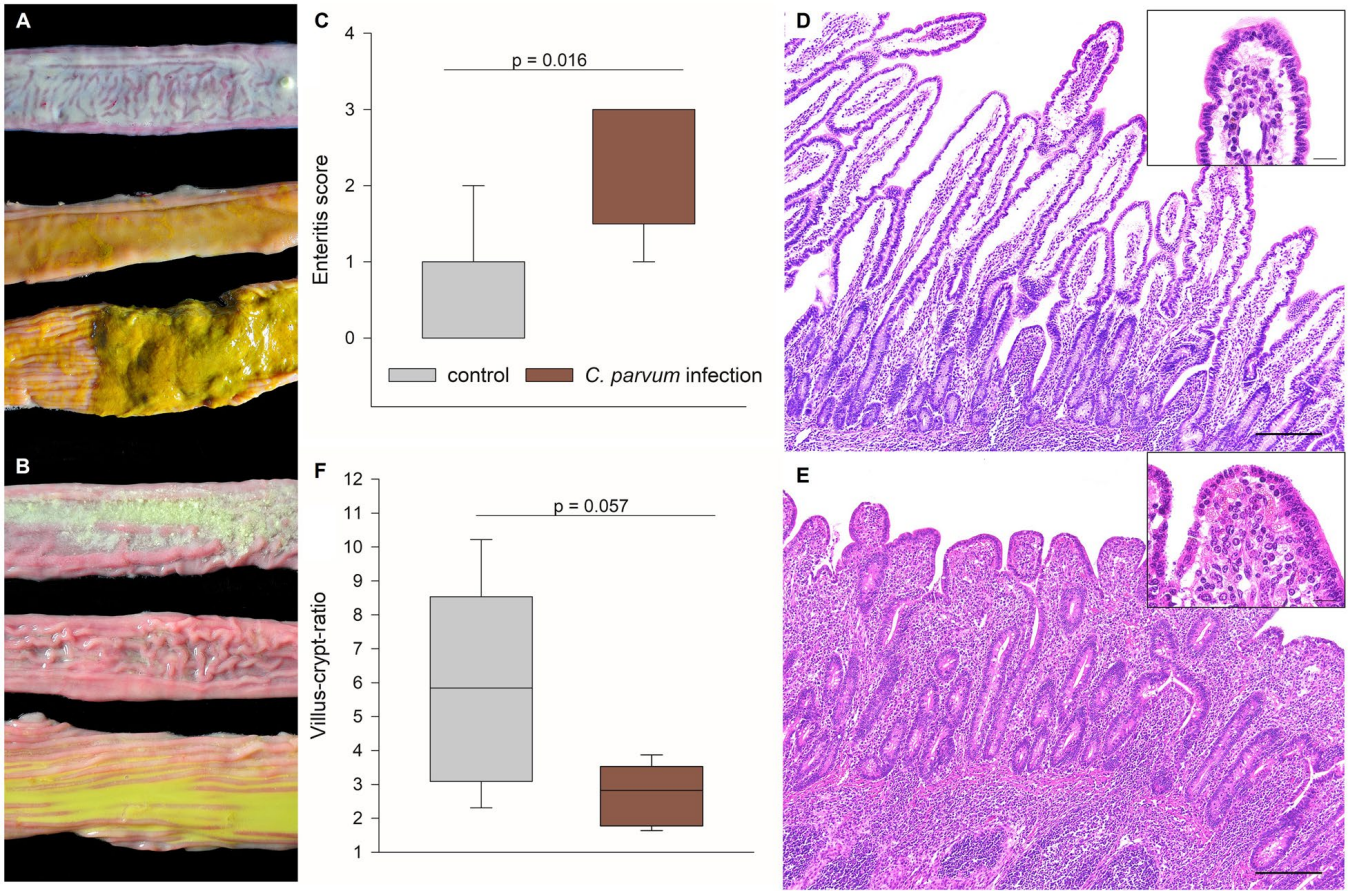
**Macroscopic and histopathologic findings**. Macroscopic differences in the consistency of the ingesta in cranial (top) and caudal small intestine (middle) and colon (bottom) of **A** control calves and **B** *C. parvum*-infected calves are most obvious in the colon. **C** Macroscopic evaluation revealed a severe, acute to subacute, diffuse, catarrhal enteritis in the *C. parvum*-infected calves (red boxes), whereas all but one calves of the control group (gray boxes) displayed no enteritis; *N* = 5, Mann-Whitney Rank Sum Test, *p* < 0.05. The main histopathological finding is a marked reduction in the small intestinal villus length in the *C. parvum*-infected (**D**) as compared to control calves (**E**); scale bar = 200 μm, hematoxylin-eosin staining. Higher magnification (inserts) displays multiple apicomplexan cysts characteristic of *C. parvum*; scale bar = 20 μm. **F** Marked villus atrophy as the histopathological correlate of the macroscopic enteritis is demonstrated by a decreased villus-crypt-ratio in the jejunum of infected calves (red boxes) compared to control calves (gray boxes). *N* = 5, Student’s *t*-test. Boxes show median and percentiles plus error bars.

The animals were euthanized 7 days pi, and autopsy was performed, including standardized sampling of the gastrointestinal tract for histopathology, immunohistochemistry, and further examinations. Macroscopical examination revealed a mild to severe, acute to subacute, diffuse, catarrhal enteritis characterized by watery, yellow ingesta in the caudal small and large intestines in the *C. parvum*-infected calves, whereas characteristic pasty, yellow ingesta were observed in all but one control animals (Figures 2A, B). There was a significant difference between the median severe grade of catarrhal enteritis in the *C. parvum*-infected group and the control group (Figure 2C; *p* < 0.05, Mann-Whitney Rank Sum Test). Histopathology revealed mild to moderate villus atrophy and fusion with numerous small, round oval, about 3 to 6 μm in diameter, apicomplexan cysts (*Cryptosporidium* spp.) in the *C. parvum*-infected calves as compared to controls (Figures 2D, E). The villus-crypt-ratio in the jejunum was decreased by over 50% from a median of 5.8 in the controls to 2.8 in the *C. parvum*-infected group (Figure 2F).

### Calves infected with ***C. parvum*** have lower glucose and higher urea plasma concentrations

**Figure 3:**
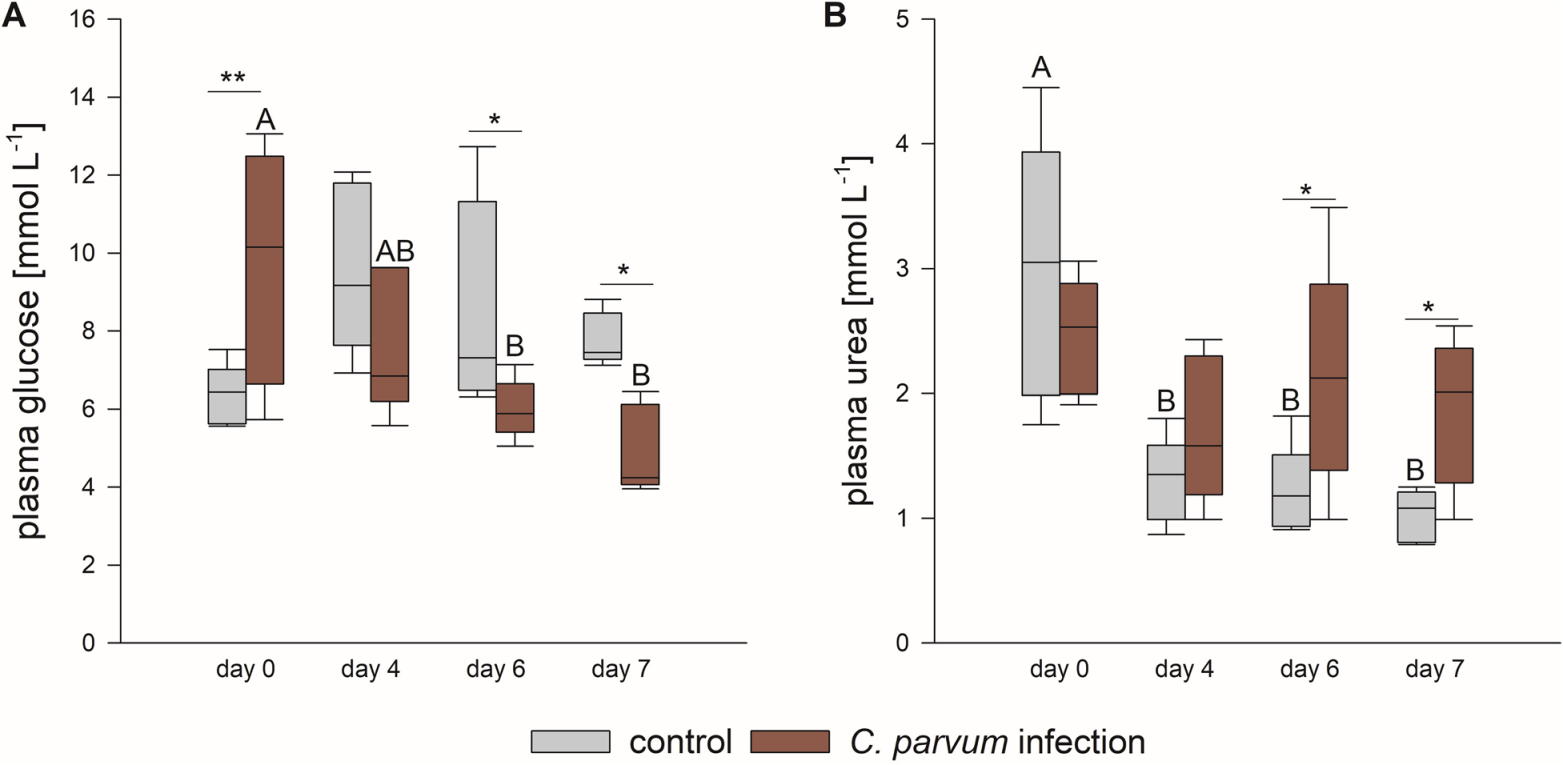
**Plasma glucose (A) and urea (B) concentrations differed significantly between the treatment groups.** Boxes show median and percentiles plus error bars; *N* = 5, *p < 0.05, **p < 0.01, different letters (A, B) indicate significant differences between time points within the same group; two-way RM ANOVA with post-hoc Holm-Sidak-test.

Before infection (day 0) and on day 4, 6 and 7 pi, blood samples were collected and analyzed for plasma concentrations of glucose and urea (Figure 3). There was a significant interaction between the time point and treatment group for both parameters (*p* = 0.002 [glucose] and *p* = 0.036 [urea], respectively; two-way RM ANOVA).

On day 0, the plasma concentration of glucose was higher in the calves dedicated to the infection group (but not yet infected) compared to the control group (*p* = 0.008). We have no definite explanation for this difference between the groups before the infection but can only speculate about a shorter time span between feeding at the farm and the blood collection or higher stress levels induced by the transport and handling in these animals by chance. Generally, plasma glucose levels appear to have a quite high variability on the first day of life in neonatal calves [36]. On days 6 and 7 pi, the infection group had a lower plasma glucose concentration compared to the control group and compared to the baseline values on day 0 (*p* < 0.05).

In contrast to that, the plasma urea concentration was significantly increased in the infected calves on days 6 and 7 pi compared to the control group (*p* < 0.05). In the control group, the plasma urea concentration was decreased significantly on days 4, 6 and 7 pi compared to day 0 (*p* < 0.05) but not in the infected animals.

### The absorption of oral glucose is decreased but more glucose is oxidized in infected calves

**Figure 4:**
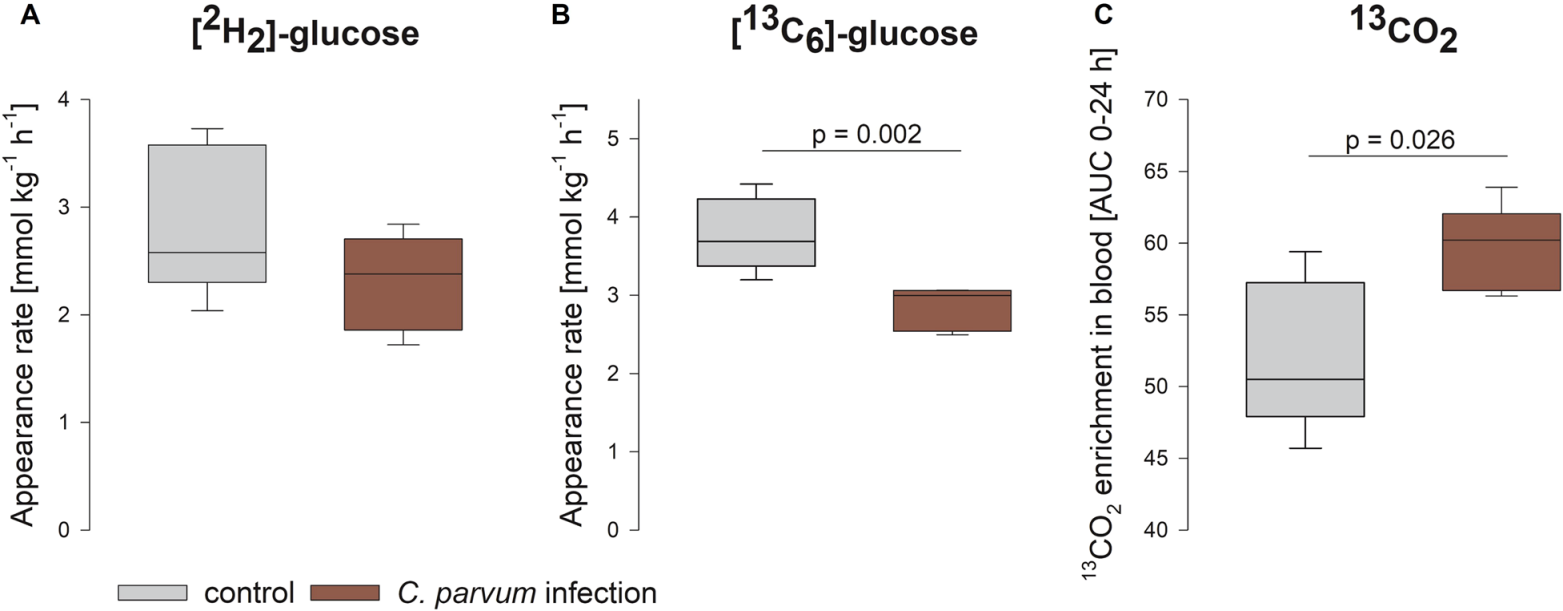
**Appearance rate of [**^**2**^**H**_**2**_**]- and [**^**13**^**C**_**6**_**]-glucose after systemic and oral application and enrichment of **^**13**^**CO**_**2**_. There was no difference in systemic glucose utilization (**A**), but a significantly higher appearance rate of orally applied [^13^C_6_]-glucose in the control group (gray boxes) compared to the infected calves (red boxes) (**B**). The appearance rate of ^13^CO_2_ was significantly higher in the infected group compared to the control group (**C**). Boxes show median and percentiles plus error bars; *N* = 5, Student’s *t*-test.

On day 6 pi, we analyzed the glucose absorption and turnover of the calves after feeding. [^2^H_2_]-labelled glucose was administered i.v. to monitor systemic glucose turnover and at the same time [^13^C_6_]-labelled glucose was given orally to track its absorption. While there was no difference in the appearance rate for [^2^H_2_]-glucose, the appearance rate of orally applied [^13^C_6_]-glucose in the systemic circulation was significantly higher in the control compared to the infected group (*p* < 0.01, Student’s *t*-test; Figures 4A, B). As a proxy for the oxidation of glucose, ^13^CO_2_ enrichment in the blood was measured and the area under the curve was calculated for 24 h after the application of the isotope [30]. This value was higher in the infected calves compared to the control group (*p* < 0.05, Student’s *t*-test; Figure 4C).

### Isolated jejunum epithelium of infected calves displays a higher activity of SGLT1 in Ussing chamber experiments

**Figure 5:**
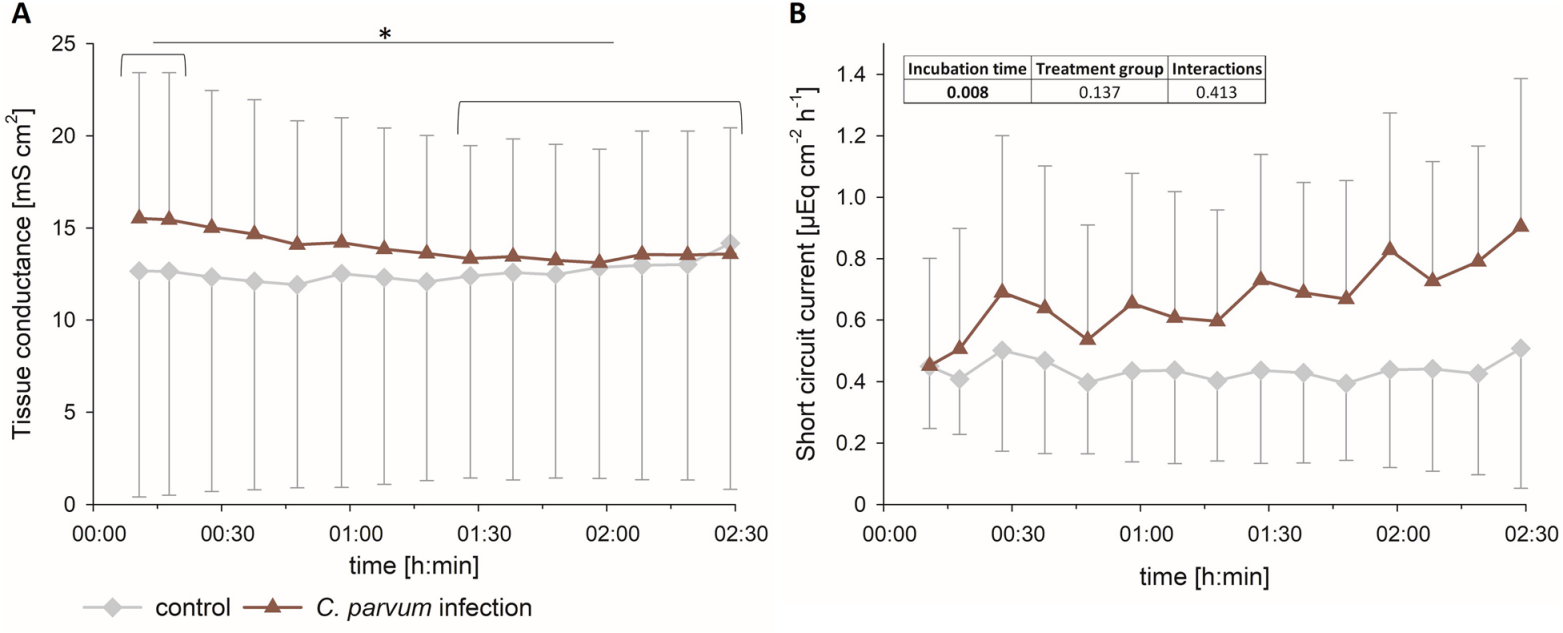
**G**_**t **_**(A) and I**_**sc **_**(B) in isolated jejunum epithelium mounted in Ussing chambers from control (gray lines) and infected calves (red lines) on day 7 pi.** Symbols represent mean values ± SD; *N* = 3 (control) and 5 (infection); different letters (A, B) indicate significant differences between the respective time points in the infected group; two-way RM ANOVA with post-hoc Holm-Sidak-test.

On day 7 pi the calves were sacrificed, and isolated jejunum epithelium was mounted in Ussing chambers to assess the transepithelial transport of glucose. Basic electrophysiological parameters are shown in Figure 5. Regarding the tissue conductance (G_t_), there was a significant interaction between the effects of incubation time and treatment group (*p* < 0.001, two-way RM ANOVA). While there was no difference between the control and the infected animals, the G_t_ was significantly higher in the first 20 min of incubation compared to the values after 80–150 min of incubation in the infected epithelia (*p* < 0.05), but not in the epithelia isolated from control animals. For the short circuit current (I_sc_), we detected no interactions between incubation time and treatment group. However, I_sc_ was significantly different over time throughout the experiment (*p* < 0.01).

**Figure 6:**
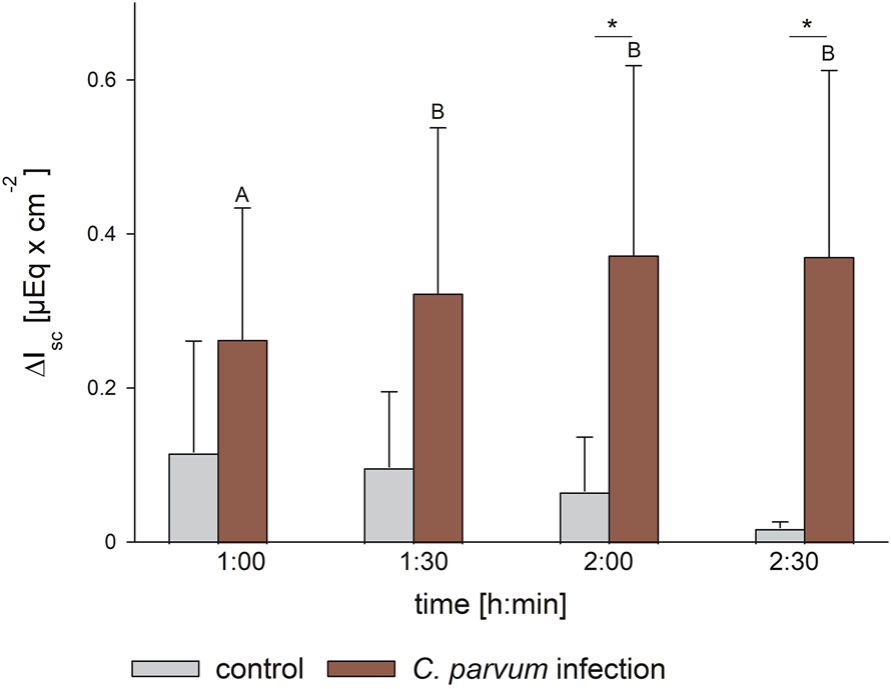
**Phlorizin sensitive I **_**sc **_**after mucosal addition of glucose in isolated jejunum epithelia mounted in Ussing chambers.** Bars show mean values ± SD; *N* = 3 (control, gray bars) or 5 (infection, red bars); different letters (A, B) indicate significant differences between the respective time points within the infected group; *p < 0.05; two-way RM ANOVA with post-hoc Holm-Sidak-test.

We measured the activity of SGLT1 by determining the phlorizin sensitive I_sc_ after mucosal addition of 2 mmol L^− 1^ glucose (ΔI_sc_). There was an interaction between the incubation time and the treatment group (*p* < 0.05, two-way RM ANOVA) and comparison within the groups revealed a significant increase of ΔI_sc_ from the first glucose challenge to the following in the infected epithelia only (*p* < 0.01). Furthermore, ΔI_sc_ was significantly higher in the infected epithelia compared to the control group after 2 and 2.5 h of incubation (*p* < 0.05; Figure 6).

### Genes for glucose metabolism, but not transport are upregulated in infected epithelia

**Figure 7:**
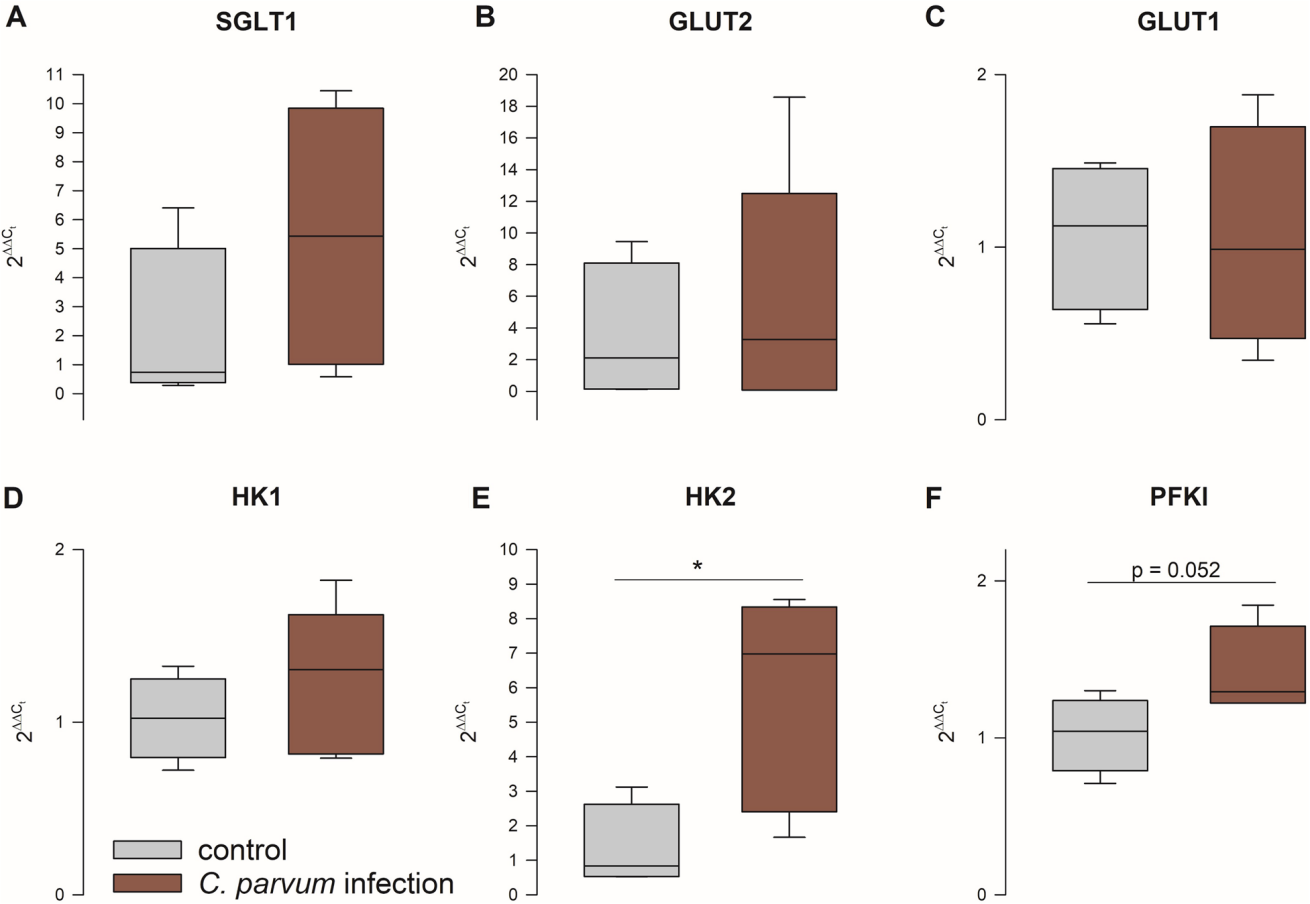
**mRNA expression of genes involved in the metabolism and transepithelial transport of glucose.** *HK2* and *PFKl* were upregulated in the infected epithelia (red boxes) compared to the control group (gray boxes) on day 7 pi. Boxes show median and percentiles plus error bars, *N* = 4 (control) and 5 (infection), Student’s *t*-test, *p < 0.05.

The mRNA expression of genes involved in the metabolism and transepithelial transport of glucose was examined by RT-qPCR (Figure 7). We found an increase in hexokinase (*HK*) 2 and phosphofructokinase (liver type, *PFKl*) in the jejunum epithelium of infected calves compared to the control group (*p* = 0.034 and *p* = 0.052, respectively, Student’s *t*-test). The expression of *HK1*, *SGLT1*, *GLUT1* and *GLUT2* was not different between the groups.

### SGLT1 and GLUT2 protein levels are not different on total protein level, but GLUT2 is enriched in the brush border membrane

**Figure 8:**
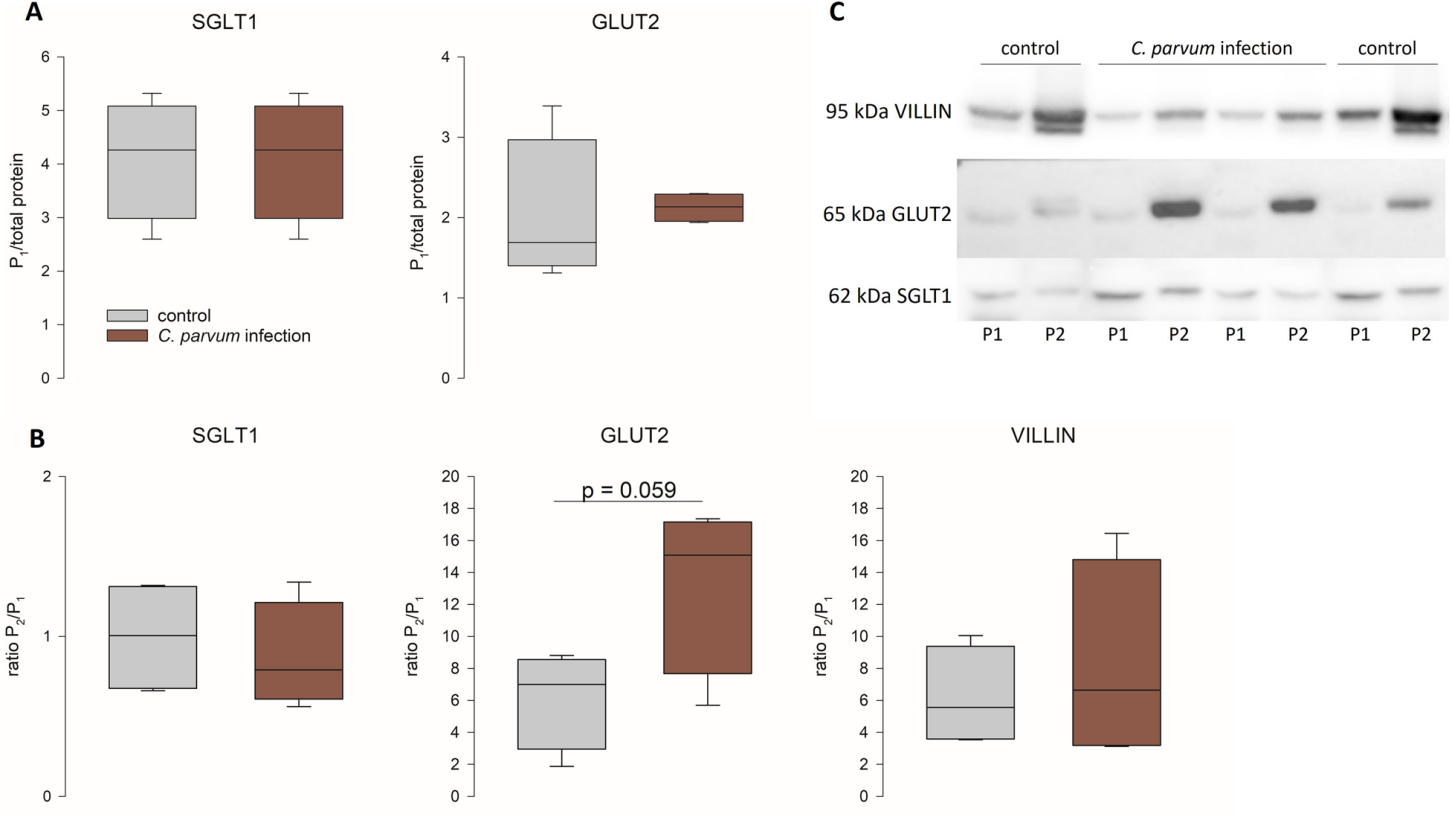
**Protein levels of SGLT1 and GLUT2 in epithelia from control (gray boxes) and infected calves (red boxes).** **A** shows the protein abundance in total protein extracts (P1) while **B** displays the protein levels in BBM preparations (P2) in relation to P1, i.e., the apical enrichment of each protein. Enrichment of VILLIN was calculated to verify the BBM preparation. Boxes show median and percentiles plus error bars, *N* = 4 (control) and 5 (infection); Student’s *t*-test. **C** shows representative blots.

There was also no difference in the overall abundance of SGLT1 and GLUT2 between infected and uninfected calves on the protein level (Figure 8A). However, Western blot analysis of brush border membrane preparations (BBM) revealed an accumulation of GLUT2 in the apical membrane (Figure 8B). This accumulation was more pronounced in infected epithelia than in control epithelia (*p* = 0.059, Student’s *t*-test). SGLT1 showed no differences between the infected and the control group and also no enrichment in the BBM. To verify the correct preparation of BBM, the enrichment of VILLIN was also calculated and resulted in similar rates in both groups (Figure 8B).

**Figure 9:**
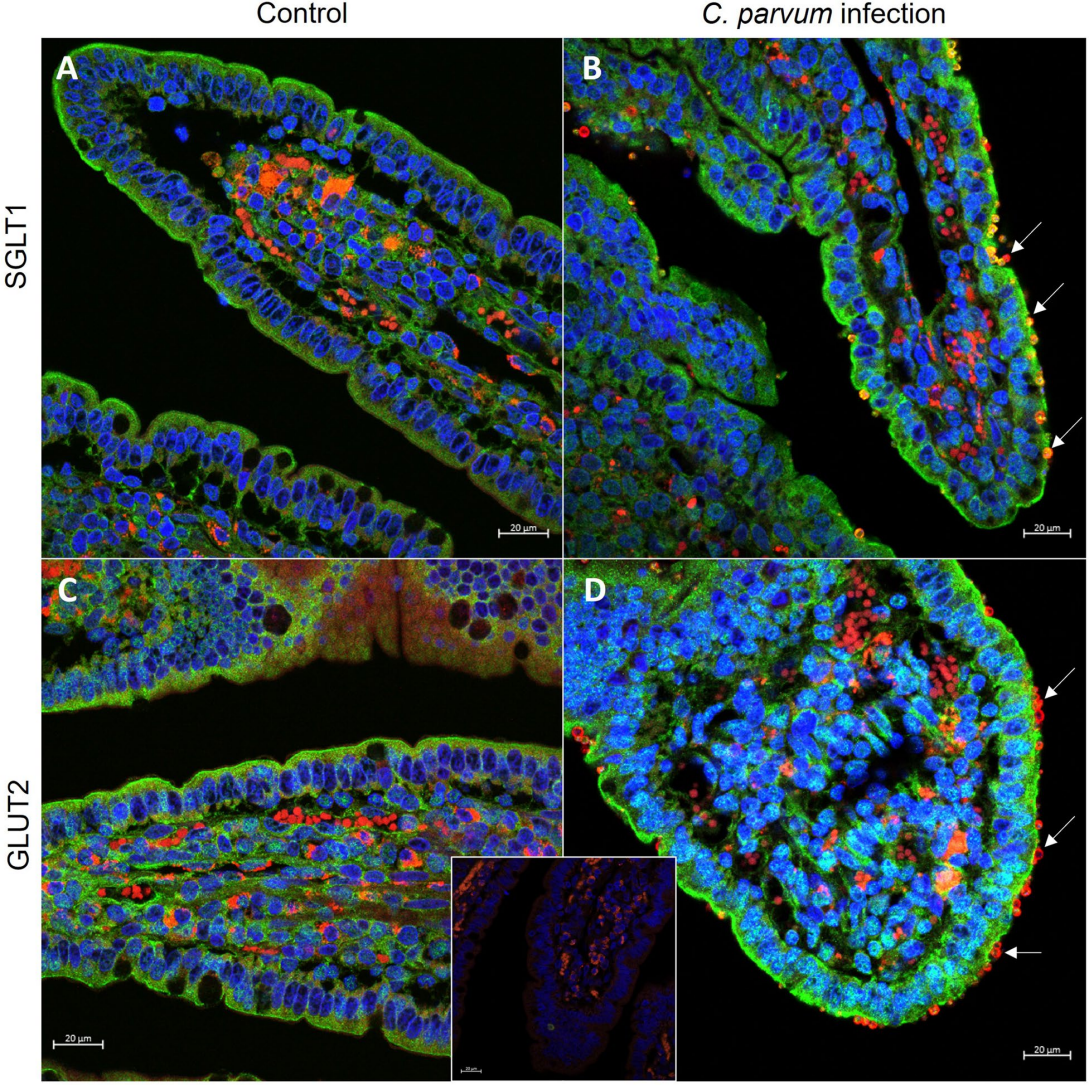
**Immunofluorescent staining of SGLT1 (A, B, green) and GLUT2 (C, D, green) in epithelia from control (A, C) and infected (B, D) animals.** White arrows indicate parasite stages attached to the epithelial cells (red staining at the outside of the villi, unspecific staining of erythrocytes inside the villi). Cell nuclei are counterstained in blue. The insert shows a control stained with secondary antibody only. Scale bars: 20 μm.

The apical localization of GLUT2 could be confirmed by immunofluorescent staining (Figure 9). The staining also demonstrates an intact epithelial lining of the intestinal villi.

## Discussion

Cryptosporidiosis is a worldwide health issue that negatively affects both human and animal welfare [1, 37]. To improve the limited therapeutic options, a better understanding of the parasitic strategies and its interaction with the host cells is needed. To our knowledge, this is the first study demonstrating the effects of *C. parvum* infection on the intestinal transepithelial transport characteristics in conjunction with systemic consequences in neonatal calves.

Glucose and galactose derived from milk lactose constitute an important energy source for neonatal calves [38, 39]. Hence, their efficient uptake is vital. However, *C. parvum* also depends on glucose for energy supply and thus the uptake of this substrate into the host cells [16]. *C. parvum* is equipped with several glucose and amino acid transporters that are supposed to mediate the uptake of these substrates from the host cell cytoplasm [13]. Previous in vitro studies indicated that the modulation of cellular glucose uptake and metabolism might be an important pathomechanism of *C. parvum* infection [14, 15]. Our results indicate an upregulation of the glucose uptake and metabolism machinery in *C. parvum* infected jejunum epithelium in vivo at simultaneously lower systemic availability of glucose.

The plasma glucose concentrations were significantly diminished 6 days pi and at the same time plasma urea concentrations increased, which might be a sign of a catabolic systemic metabolism due to an energy deficit in the infected calves. Although the body mass of the calves was not significantly different on day 6 pi, the calves appeared to gain less weight compared to the healthy control group and it has been described earlier that calves with signs of diarrhea in the first months of life had lower weight gain on the long run [40]. A reason why the difference was not more distinct in our study might have been the restricted feeding limiting the growth of the control group and the low animal number, which is a general limitation of this study.

The lower plasma glucose levels in the infected group are in line with the findings of the tracer experiments conducted on day 6 pi. While the systemic metabolism of glucose appeared to be similar in both groups, as shown by comparable appearance rates of the tracer injected intravenously, the appearance rate in the systemic circulation of the tracer applied orally was significantly lower in the infected calves compared to the control group. This indicates that the systemic glucose usage beyond the splanchnic area was not affected by the infection, but the absorption, i.e., the transfer of glucose from the intestinal lumen into the circulation, was significantly reduced by the infection. There might be a loss of glucose in the gastrointestinal tract due to impaired apical uptake, cellular transit or extrusion, or glucose is intercepted by the intracellular parasite and/or submucosal immune cells. The increased enrichment of ^13^CO_2_ in the blood plasma suggests that more glucose was oxidized in the splanchnic area of the infected calves. This assumption is further supported by the higher mRNA expression of glycolysis enzymes in the mucosa.

To elucidate the transepithelial transport of glucose in more detail, we conducted Ussing chamber experiments. In general, we found only slight differences in the electrophysiological parameters between the groups, indicating that the tissue integrity was not substantially disrupted by the infection. This is in accordance with studies in rat, mouse and human models, where G_t_ was either not affected by *C. parvum* infection as well or even decreased compared to an uninfected control [41–43], whereas in vitro experiments using T84 cells showed an increased tissue permeability after infection [44]. This discrepancy emphasizes the importance of in vivo studies in the actual host and may also indicate differences in epithelial integrity during the course of infection. However, an increase in phlorizin sensitive I_sc_ indicates that SGLT1 is more active in infected epithelia. Interestingly, the glucose-evoked current increased in the infected epithelia but decreased in the control group over time, enhancing the difference. We can only speculate about the reasons for this development. It may be possible that with the epithelium being transported in ice-cold buffer solution, the transporter activity was re-established to its full extent only after a prolonged incubation period or there might be a connection between the infection and a higher sensitivity of SGLT1 to stimuli like (mechanical) stress or luminal glucose. Our results are contradictory to a reportedly reduced glucose-stimulated Na^+^ absorption across the jejunum epithelium of piglets and suckling rats infected with *C. parvum* [41, 45]. This difference might root in the time point during infection chosen for studying the electrogenic glucose transport. While these previous studies tested glucose uptake at the peak of infection, we performed the Ussing chamber experiments on day 6 pi whereas the oocyst shedding peaked at day 4 pi in our setup. Moreover, the glucose-stimulated current that was measured in other studies is not necessarily identical with SGLT1 activity as there might be secondary effects on other transport mechanisms. Therefore, we used the inhibitor phlorizin to determine the SGLT1 activity specifically. With a likely explanation for the reduced glucose-induced current being the loss of villus epithelium [45], our findings could mirror an ongoing re-epithelialization to repair damage that has been inflicted earlier. Pathohistological measurements indicated villus stunting but also revealed an intact epithelium covering the villi. Furthermore, protein levels of VILLIN were similar in both groups in the apical membranes. Hence, there is some evidence supporting this assumption of new cells with a high absorptive activity. However, it must be kept in mind that these findings have been obtained ex vivo. Therefore, there might be an effect of the epithelial preparation or incubation on transepithelial transport, which could also account for diverging results. Furthermore, there might be species-specific differences, although a completely opposite regulation between pigs, rats and calves seems to be rather unlikely.

The increased activity of SGLT1 was not matched on the gene and protein expression levels of SGLT1, and its numbers in the BBM were also not increased. Thus, either this functional observation made ex vivo was caused by post mortal processes and cannot be transferred to the in vivo situation or it must be mediated by a posttranslational modification of transport proteins already available in the membrane, rendering them more active. The latter would mean that the apical uptake of glucose is enhanced while based on the tracer appearance rates, less glucose is being delivered into the systemic circulation. Moreover, the enrichment of GLUT2 in the BBM indicates a recruitment of this facilitative glucose transporter to the apical membrane, thereby additionally improving the uptake capacities for glucose irrespective of SGLT1. An apical localization of GLUT2 is controversial but has been shown in neonatal calves and early-weaned piglets before [22, 46]. Our results confirmed not only that GLUT2 is generally present in the apical membrane of jejunum epithelium of neonatal calves but also revealed that its recruitment is subject to regulatory influences on posttranslational, but not on gene expression level.

This regulation might originate from the enterocytes attempting to compensate for the increased energy demand due to the parasitic nutritional competition and/or inflammatory state or from the parasite manipulating its host to optimize energy supply. Furthermore, an apical enrichment of GLUT2 could also be a sign for less differentiated and thus less clearly polarized enterocytes replacing the damaged epithelium after release of the oocysts, supporting the re-epithelialization hypothesis. Our study cannot solve this question, but it may offer some hints. While an enhanced luminal uptake of glucose may serve both the host and the parasite, the upregulation of *HK2* and *PFKl* along with the increased enrichment of ^13^CO_2_ strongly suggest an upregulation of the enterocytes’ glycolytic capacities and thus a cellular counter regulation in infected calves. This would subsequently increase the intracellular phosphorylation of glucose and thereby enhance glucose utilization by the enterocytes, increase the concentration gradient for its uptake via GLUT2 and simultaneously prevent its extrusion to the systemic circulation as demonstrated in the tracer experiments. Hence, there is a large body of evidence for an increased epithelial energy demand. Findings of an increased cellular glutaminolysis in infected HCT-8 cells in vitro support this [15].

In conclusion, our results provide evidence for a nutritional competition of *C. parvum* with the infected enterocytes resulting in upregulation of glucose uptake and metabolism in these cells. However, while this might be a first important measure to ensure epithelial survival and thus integrity, it is apparently not sufficient to provide the organism with energy. Less glucose is transferred from the gut lumen to the systemic circulation leading to decreased plasma glucose concentrations, which may pave the way for complications such as secondary infections. Although infected calves obviously need more energy, no therapeutic recommendations can be derived from these observations yet, since it is unclear, whether parasite or host will benefit most from oral or systemic supplementation.

## Supplementary Information

**Additional file 1: **Clinical parameters.

## Data Availability

The datasets used and/or analysed during the current study are available from the corresponding author on reasonable request.
