# Supplementary material for: Cryptosporidium parvum competes with the intestinal epithelial cells for glucose and impairs systemic glucose supply in neonatal calves
Source: Vet Res. 2023 May 3;54:40. doi: 10.1186/s13567-023-01172-y (PMC10156424; doi:10.1186/s13567-023-01172-y)
Supplement: Supplementary file 1 — Additional file 1: Clinical parameters. [file 13567_2023_1172_MOESM1_ESM.docx]

**Additional file 1 Clinical parameters**

|  |  | **Control** | | ***C. parvum* infection** | |
| --- | --- | --- | --- | --- | --- |
|  |  | **mean** | ***SD*** | **mean** | ***SD*** |
|  |  |  |  |  |  |
| **Body weight [kg]** | **day 0** | 44.60 | *3.32* | 41.70 | *5.12* |
|  | **day 6** | 50.20 | *6.13* | 43.80 | *4.38* |
|  |  |  |  |  |  |
| **internal body temperature [°C]** | **day 0** | 38.48 | *0.26* | 38.64 | *0.22* |
|  | **day 1** | 38.86 | *0.39* | 38.74 | *0.80* |
|  | **day 2** | 38.82 | *0.76* | 38.92 | *0.29* |
|  | **day 3** | 39.18 | *0.39* | 38.82 | *0.36* |
|  | **day 4** | 39.30 | *0.42* | 38.74 | *0.38* |
|  | **day 5** | 38.78 | *0.44* | 39.26 | *0.15* |
|  | **day 6** | 39.02 | *0.28* | 38.66 | *0.83* |
|  | **day 7** | 39.06 | *0.41* | 39.13 | *0.55* |
|  |  |  |  |  |  |
| **Oocysts [× 10^5^ / g faeces]** | **day 0** |  |  | 0.00 | *0.00* |
|  | **day 1** |  |  | 0.00 | *0.00* |
|  | **day 2** |  |  | 0.40 | *0.89* |
|  | **day 3** |  |  | 4.18 | *4.90* |
|  | **day 4** |  |  | 29.49 | *24.02* |
|  | **day 5** |  |  | 15.30 | *9.26* |
|  | **day 6** |  |  | 5.40 | *2.87* |
|  | **day 7** |  |  | 3.46 | *1.77* |
|  |  |  |  |  |  |
| **faeces score** |  | **median** | ***range*** | **median** | ***range*** |
|  | **day 0** | 0.0 | *0 - 2* | 0.0 | *0.0* |
|  | **day 1** | 0.0 | *0 - 1* | 0.0 | *0 - 3* |
|  | **day 2** | 0.0 | *0 - 1* | 0.0 | *0 - 2* |
|  | **day 3** | 0.0 | *0 - 1* | 2.0 | *0 - 2* |
|  | **day 4** | 0.0^a^ | *0 – 0.5* | 2.0^b^ | *1.5 - 2* |
|  | **day 5** | 0.5^a^ | *0 – 1.5* | 2.0^b^ | *2.0* |
|  | **day 6** | 0.5^a^ | *0 - 1* | 2.0^b^ | *2.0* |
|  | **day 7** | 1.0^a^ | *0 - 1* | 2.0^b^ | *2.0* |
